# Infectious Diseases: Pathophysiology, Diagnostics and Prevention

**DOI:** 10.3390/ijms17091464

**Published:** 2016-09-02

**Authors:** Susanna Esposito

**Affiliations:** Pediatric Highly Intensive Care Unit, Department of Pathophysiology and Transplantation, Università degli Studi di Milano, Fondazione IRCCS Ca’ Granda Ospedale Maggiore Policlinico, 20122 Milan, Italy; susanna.esposito@unimi.it; Tel.: +39-02-5503-2498; Fax: +39-02-5032-0206

Infectious diseases occur very frequently in children and adults. Novel diagnostic methods have permitted us to expand our knowledge on their epidemiology and pathophysiology [1]. Emerging pathogens have appeared in recent years, raising alerts among health authorities. Moreover, new anti-infective treatments have been discovered, and it is extremely important to know when to use them to avoid abuse and misuse of these new drugs because of possible negative antimicrobial resistance outcomes [2]. Furthermore, new vaccines have appeared on the market, and it is essential that practitioners be informed of their impact on public health [3]. For these reasons, the *International Journal of Molecular Sciences* has decided to dedicate a Special Issue to the pathophysiology, diagnostics and prevention of infectious diseases [4]. A total of 22 papers consisting of 12 original research studies, 9 reviews and 1 case report have been published in the Special Issue, as detailed in Table 1. This Special Issue has the main goal of presenting recent advances in different aspects of infectious diseases, covering pediatric and adult issues as well as different pathogens.

Nine manuscripts (five original research studies, three reviews and one case report) discussed bacterial infections, showing the significant impact of these infections in terms of disease burden among all age groups as well as advances in diagnostic and therapeutic approaches.

In a 10-year retrospective study, Pugni et al. showed the burden of neonatal septic shock in a neonatal intensive care unit and demonstrated advantages in management with a reduction in the mortality rate through the use of exchange transfusion in addition to standard therapy [5]. According to the authors’ conclusions, the lack of adverse events should encourage the use of this procedure in the treatment of neonates with septic shock.

Castellazzi et al. provided clinicians with an update on recent controversies and advances regarding the management of acute osteomyelitis and septic arthritis in children [6]. In recent years, the emergence of bacterial species resistant to commonly used antibiotics that are particularly aggressive highlights the need for further research to optimize treatment approaches and to develop new molecules able to fight the war against acute osteoarticular infection in pediatric patients.

Krone et al. tested the feasibility of using dried saliva spots (DSS) for studies on pneumococcal carrier states [7]. Saliva samples from children and pneumococcus-spiked saliva samples from healthy adults were applied to paper, dried and stored, with and without desiccant, at temperatures ranging from −20 to 37 °C for up to 35 days. DNA extracted from DSS was tested with quantitative-polymerase chain reaction (PCR) specifically for *Streptococcus pneumoniae*. The authors showed that there were no differences in the results when spiking saliva with varied pneumococcal strains. The collection of saliva can be particularly useful in surveillance studies conducted in remote settings, as it does not require trained personnel and DSS can withstand various transportation conditions.

Ieni et al. discussed the role of cellular morphological effectors of innate immunity during *Helicobacter pylori* infection and gastric carcinogenesis [8], whereas Liu et al. analyzed the different immunogenicity of outer membrane proteins from *Salmonella* mutants [9] and Gallo et al. reported a case of fatal sepsis caused by *Campylobacter jejuni* in a 76-year-old Caucasian man with non-Hodgkin’s lymphoma [10].

Interestingly, three of the manuscripts on bacterial infections analyzed topics related to different aspects of tuberculosis, highlighting that *Mycobacterium tuberculosis* remains a global health challenge. Korbe et al. presented evidence of the mechanisms by which *M. tuberculosis* manipulates the protective immune response to become a pathological productive infection [11].

Galli et al. presented results from a multicenter study conducted in 27 pediatric hospitals, pediatric wards and public health centers in Italy using a standardized form covering the period of time between 1 January 2010 and 31 December 2012 [12]. The results showed that improving the surveillance of childhood tuberculosis is important for public health care workers and pediatricians. Moreover, it was demonstrated that a non-negligible proportion of children had drug-resistant tuberculosis and were treated with second-line drugs, most of which are off-label for the pediatric age range.

Finally, Sotgiu et al. performed a systematic review to evaluate the efficacy, safety and tolerability of carbapenems in treatment of multi-drug-resistant tuberculosis [13]. Two studies on ertapenem, one on imipenem and four on meropenem, all published between 2005 and 2016, were identified. The treatment success was greater than 57% in five studies with culture conversion rates between 60% and 94.8%. The safety and tolerability of carbapenems appeared to be very good, with the proportion of adverse events attributable to carbapenems below 15%.

Seven manuscripts (all original research) discussed viral infections, showing how molecular diagnostics have permitted us to expand our knowledge on viral epidemiology and pathogenesis.

Tramuto et al. reconstructed the phylogenetic relationships and genetic diversity of the hemagglutinin gene sequences of 197 influenza B strains circulating in both southern (Sicily) and northern (Liguria) Italy between 2010 and 2015 [14]. Phylogenetic analysis showed clusters in the B/Victoria clade 1A/1B (*n* = 29, 14.7%) and the B/Yamagata clades 2 (*n* = 112, 56.8%) and 3 (*n* = 56, 28.4%). Both influenza B lineages were found to co-circulate during the study period, providing evidence to support the adoption of quadrivalent influenza vaccines.

Esposito et al. compared the last version of the Respiratory Virus Panel (RVP) Fast assay for human adenovirus (hAdv) detection with specific real-time PCR, showing that the RVP Fast V2 assay has important limitations for the detection of hAdv and cannot be used either to evaluate whether hAdvs are the main etiologic agent responsible for an outbreak or when epidemiological studies are performed [15].

Gagliardi et al. evaluated the prevalence, type and clinical impact of specific viral genomes in endomyocardial biopsies (EMBs) collected between 2001 and 2013 from 63 children admitted for acute heart failure (median age 2.8 years) [16]. Presence of a virus-positive EMB was associated with improvement in cardiac function over time and better long-term prognosis.

Bozzola et al. described varicella skin complications in hospitalized immunologically healthy children over a nine-year period, showing that they occurred in 2.6%–41.2% of cases [17].

Vorsters et al. investigated the use of urine samples, which can be collected at home, instead of cervical samples, for follow-up of a human papillomavirus (HPV) intervention trial, demonstrating that urine sampling may be a valid alternative to cervical samples [18].

Latronico et al. showed that the decrease of matrix metalloproteinases (MMP-9) following hepatitis C (HCV) protease inhibitor treatment suggests a positive effect on the reduction of liver inflammation [19].

Focà et al. assessed neurocognitive profiles of newly detected HIV-infected patients, showing that complete neuropsychological screening must be recommended for all patients at the time of HIV-infection diagnosis [20].

Four manuscripts (all reviews) discussed emerging infectious-related diseases, highlighting the potential role of different pathogens in immune-mediated disorders.

Rigante et al. showed that early and definite determination of likely intravenous immunoglobulin non-responders who require additional therapies to reduce the development of coronary artery involvement in children with Kawasaki syndrome is still a challenge [21].

Torreggiani et al. summarized infectious and non-infectious causes of recurrent fever in pediatric patients [22], whereas Principi and Esposito discussed recent concepts regarding pediatric discitis and spondylodiscitis [23] and Di Pasquale et al. reviewed non-intensive care unit–acquired pneumonia [24].

Two manuscripts (both reviews) discussed vaccines, focusing on targets that, in recent years, have been considered as priorities for vaccine use.

De Martino discussed the importance of maternal immunization, highlighting that strong communication strategies are effective for reducing parental vaccine hesitancy and approvals by regulatory agencies for the use of vaccines during pregnancy are needed [25].

Finally, Orsi et al. summarized the available evidence from Italy and Liguria regarding pneumococcal carriage, the burden of community-acquired pneumonia and lower respiratory tract infections and the on-field effectiveness of 13-valent pneumococcal conjugate immunization in adults and the elderly [26].

Overall, the 22 contributions published in the Special Issue illustrate the advances in the field of infectious diseases in recent years due to new diagnostic possibilities, the involvement of bacteria and viruses in acute diseases and in different medical disciplines and the importance of prevention with effective and safe vaccines. We would like to thank all the authors who contributed to the Special Issue, and we remain hopeful that adequate knowledge of how to diagnose, treat and prevent infectious diseases will help to contain global morbidity and mortality.

## Figures and Tables

**Table 1:** Summary of papers in the Special Issue “Infectious diseases: pathophysiology, diagnostics and prevention”, arranged by topic.

Authors	Title	Topics	Type
Pugni et al. [5]	Exchange transfusion in the treatment of neonatal septic shock: A ten-year experience in a neonatal intensive care unit	Bacteria	Original research
Castellazzi et al. [6]	Update on the management of pediatric acute osteomyelitis and septic arthritis	Bacteria	Review
Krone et al. [7]	Dried saliva spots: A robust method for detecting *Streptococcus pneumoniae* carriage by PCR	Bacteria	Original research
Ieni et al. [8]	Morphological and cellular features of innate immune reaction in *Helicobacter pylori* gastritis: A brief review	Bacteria	Original research
Liu et al. [9]	Immunogenicity and cross-protective efficacy induced by outer membrane proteins from *Salmonella typhimurium* mutants with truncated LPS in mice	Bacteria	Original research
Gallo et al. [10]	*Campylobacter jejuni* fatal sepsis in a patient with Non-Hodgkin’s lymphoma: Case report and literature review of a difficult diagnosis	Bacteria	Case report
Korb et al. [11]	*Mycobacterium tuberculosis:* Manipulator of protective immunity	Bacteria	Review
Galli et al. [12]	Pediatric tuberculosis in Italian children: Epidemiological and clinical data from the Italian Register of Pediatric Tuberculosis	Bacteria	Original research
Sotgiu et al. [13]	Carbapenems to treat multidrug and extensively drug-resistant tuberculosis: A systematic review	Bacteria	Review
Tramuto et al. [14]	The molecular epidemiology and evolutionary dynamics of influenza B virus in two Italian Regions during 2010–2015: The experience of Sicily and Liguria	Viruses	Original research
Esposito et al. [15]	Identification of human Adenovirus in respiratory samples with Luminex Respiratory Virus Panel Fast V2 assay and real-time polymerase chain reaction	Viruses	Original research
Gagliardi et al. [16]	The impact of specific viruses on clinical outcome in children presenting with acute heart failure	Viruses	Original research
Bozzola et al. [17]	Varicella skin complications in childhood: A case series and a systematic review of the literature	Viruses	Original research
Vorsters et al. [18]	Long-term follow-up of HPV infection using urine and cervical quantitative HPV DNA testing	Viruses	Original research
Focà et al. [19]	Screening for neurocognitive impairment in HIV-infected individuals at first contact after HIV diagnosis: The experience of a large Clinical Center in Northern Italy	Viruses	Original research
Latronico et al. [20]	Liver fibrosis in HCV monoinfected and HIV/HCV coinfected patients: Dysregulation of matrix metalloproteinases (MMPs) and their tissue inhibitors TIMPs and effect of HCV protease inhibitor	Viruses	Original research
Rigante et al. [21]	Critical overview of the risk scoring systems to predict non-responsiveness to intravenous immunoglobulin in Kawasaki syndrome	Emerging infectious-related diseases	Review
Torreggiani et al. [22]	Recurrent fever in children	Emerging infectious-related diseases	Review
Principi & Esposito [23]	Infectious discitis and spondylodiscitis in children	Emerging infectious-related diseases	Review
Di Pasquale et al. [24]	Non-intensive care unit acquired pneumonia: A new clinical entity?	Emerging infectious-related diseases	Review
De Martino [25]	Dismantling the taboo against vaccines in pregnancy	Vaccines	Review
Orsi et al. [26]	Pneumococcus and the elderly in Italy: A summary of available evidence regarding carriage, clinical burden of lower respiratory tract infections and on-field effectiveness of PCV13 vaccination	Vaccines	Review
